# Assessment of Antioxidant Properties of Classic Energy Drinks in Comparison with Fruit Energy Drinks

**DOI:** 10.3390/foods9010056

**Published:** 2020-01-07

**Authors:** Dariusz Nowak, Michał Gośliński

**Affiliations:** Department of Nutrition and Dietetics, Faculty of Health Sciences, Ludwik Rydygier Collegium Medicum in Bydgoszcz, Nicolaus Copernicus University in Toruń, Dębowa 3, 85-626 Bydgoszcz, Poland; m.goslinski@cm.umk.pl

**Keywords:** energy drinks, antioxidant properties, vitamin C, sugar

## Abstract

Energy drinks (EDs) contain sugar, caffeine, and other bioactive compounds. Recently, new types of EDs, enriched with fruit juices, natural pigments, and plant extracts, have been launched in the market. The objective of this study was to investigate the composition and antioxidant properties of the most popular classic and fruit EDs. The study was carried out including 24 of the most popular energy drinks (classic and fruit EDs). The composition of EDs, especially caffeine and sugar, and antioxidant properties (antioxidant capacity, total polyphenols, total anthocyanins, vitamin C) were analyzed. Energy drinks with added fruit juice or natural pigments had a significantly higher (*p* ≤ 0.05) antioxidant capacity compared to classic energy drinks. Fruit EDs had a higher concentration of polyphenols and vitamin C. In some fruit EDs, slight amounts of anthocyanins were found. Generally, EDs are not a rich source of polyphenols and are not distinguished by high antioxidant capacity. However, fruit energy drinks and/or ones with added natural pigments have much better antioxidant properties than classic EDs. Both classic and fruit EDs contain a large amount of caffeine and sugar, therefore, it would be advisable not to drink large amounts of EDs for possible related health issues.

## 1. Introduction

Energy drinks (EDs) are very popular among adolescents and young adults. A classic ED usually contains water, sugar, pigments, acidity regulator (e.g., citric acid or sodium citrate), and caffeine as well as other bioactive compounds, such as taurine, guarana extract, inositol, glucuronolactone, vitamins, mostly B ones (niacin, pantothenic acid, vitamins B_6_ and B_12_) [1,2,3]. Some EDs also have a preservative in their composition, e.g., sodium benzoate or potassium sorbate. EDs are also sold as sugar-free drinks or ones with added fruit juice or natural pigments (e.g., anthocyanins, carotenoids). A typical ED contains about 320 mg/Kg of caffeine, and other ingredients, which may have negative effects on human health, come in different amounts [4]. Caffeine is the substance whose excessive and long-term consumption is well known to raise the blood pressure and pulse rate, as well as causing higher aggregation of blood platelets and inferior function of the endothelium [5,6,7,8]. On the other hand, some studies indicate that caffeine intake (below 600 mg/day) causes mild, transient and reversible, cardiovascular effects, without lasting adverse effects [9]. Other researchers have shown that acute ingestion of coffee may result in favorable changes to markers of cardiometabolic health [10]. Moderate caffeine intake should also not affect bone mineral density (BMD) [11]. Furthermore, excessive consumption of energy drinks due to the interaction of caffeine and sugar causes adverse changes in cardiovascular system [12,13].

The literature provides ample data to support the antioxidant capacity of fruit, vegetables, and fruit juices. The health beneficial effects of fruits and fruit juices are mainly attributed to the presence of bioactive compounds such as polyphenols, mostly flavonoids (including anthocyanins), carotenoids, ascorbic acid, etc. [14,15,16]. It has not been verified yet whether energy drinks have sufficient antioxidant capacity to reduce oxidative stress. It is known that some ingredients, like taurine or ginseng, if present in adequate amounts, can demonstrate antioxidant properties [17,18].

The intense marketing of producers of energy drinks influences the nutritional behavior of adolescents and young adults. The consumption of energy drinks is growing. On the other hand, we know more about the health risks associated with the consumption of energy drinks. High content of caffeine and sugar is a problem, but on the other hand, in new versions of these drinks, there are fruit juices, vitamins, and plant extracts. The presence of polyphenolic compounds or vitamin C in EDs enriched with fruit juices can enhance the antioxidant capacity of these beverages, but this assumption needs verification. What is lacking as well is the research demonstrating whether other additives in a typical ED have antioxidant properties. Therefore, the objective of this study has been to analyze the antioxidant properties of fruit energy drinks and to compare them with classic EDs.

## 2. Materials and Methods

### 2.1. Materials

The study comprised 24 types of EDs purchased in local supermarkets. Ten samples were EDs with added fruit juice or natural pigments, e.g., anthocyanins (fruit EDs), while 14 samples were popular, classic EDs (Table 1). The classic EDs (C1–C14) most often contained 320 mg/Kg of caffeine (except C11), 100–110 g/Kg of sugar (saccharose), or glucose-fructose syrup (except C12 and C13, i.e., sugar-free EDs, with aspartame, acesulfame K, sucralose), acidity regulator (citric acid, sodium citrate), carbon dioxide, taurine, inositol, pigments (E150c, E150d, and E101), flavorings, sometimes guarana and glucuronolactone (only C11), as well as group B vitamins (most often niacin, pantothenic acid, vitamins B_6_ and B_12_). Besides these ingredients, fruit EDs added with different types of fruit juices contained slightly more sugar, i.e., 110–150 g/Kg (except F8), and other compounds such as citric or malic acid and natural pigments (anthocyanins or carotenoids).

In addition, fruit EDs contained often ginseng extract and guarana (details in Table 1). Prior to analyses, the EDs samples were stored in original containers (cans) at room temperature.

### 2.2. Methods

The pH of the tested samples of EDs was measured with a glass electrode (Hanna Instruments, Olsztyn, Poland) at room temperature.

### 2.3. Soluble Solids Content

The soluble solids content of samples was measured with a laboratory refractometer RL-3 (PZO, Warsaw, Poland), at 20 °C, similarly to the method used by Rubio-Arraez et al. [19].

### 2.4. Analysis of Vitamin C

The total vitamin C content was determined according to Polish Norm PN-A-04019:1998 [20] as previously described by Hallmann et al. [21]. A weighed juice sample was extracted in 2.0% oxalic acid. The solution was filtered. The filtrate was collected and then titrated with the 2.6-dichlorophenyloindophenol (Avantor Performance Materials, Gliwice, Poland) [20,21]. The results were expressed as milligrams of ascorbic acid (AA) per kilogram of sample.

### 2.5. Antioxidant Capacity by DPPH Assay

The antioxidant capacity of EDs was determined by a modified Yen and Chen [22] method, using 0.1 mol/L methanol solution of 1,1-diphenyl-2-picrylhydrazyl (DPPH, Sigma-Aldrich, St. Louis, MO, USA). This method is widely used to test the antioxidant capacity of fruit, vegetables, juices, and beverages. The advantages of a DPPH assay were previously described [23,24,25]. A 0.1 mL of sample was added to 2.9 mL DPPH solution and mixed. The absorbance was measured on a Hitachi U-1900 spectrophotometer (Hitachi, Tokyo, Japan) at 517 nm after 30 min of incubation at room temperature in the dark. Quantification was performed using a Trolox standard curve. The antioxidant capacity of the samples was expressed as milligrams of Trolox equivalents (Tx) per liter of sample.

### 2.6. Antioxidant Capacity by ABTS Assay

The antioxidant capacity of EDs was determined by a Re et al. [26] method with small modifications. In the ABTS method, 2,2′-azinobis-(3-ethyl-benzothiazoline-6-sulfonic acid) diammonium salt (ABTS, Sigma-Aldrich, St. Louis, MO, USA) and potassium persulfate solutions were mixed and stored overnight at room temperature in the dark for 12–16 h. ABTS solution was diluted with methanol to an absorbance of 0.70 ± 0.02 at 734 nm.

After addition of 1.0 mL of diluted ABTS solution (A734 nm = 0.700 ± 0.020) to 0.01 mL of antioxidant compounds or Trolox standards in methanol, the absorbance was measured at 734 nm against methanol after 1 min. Quantification was performed using a Trolox standard curve. The antioxidant capacity of the samples was expressed as milligrams of Tx per liter of sample.

### 2.7. Total Polyphenols by Folin–Ciocalteu Assay

The total polyphenols (TP) of the EDs were determined by the Folin–Ciocalteu assay [27]. First, 0.3 mL of sample was added to a 10-mL capacity tube, next 0.05 mL 2 mol/L Folin–Ciocalteu reagent (Sigma-Aldrich, St. Louis, MO, USA) and 0.5 mL 20% sodium carbonate solution were added. The mixture was diluted by addition of 4.15 mL distilled water and mixed. The absorbance was measured on a Hitachi U-1900 spectrophotometer at 765 nm after 30 min incubation in the dark at room temperature. A calibration curve was performed with gallic acid. The results were expressed as milligrams of gallic acid equivalents (GAE) per liter of sample.

### 2.8. Total Anthocyanins 

Total anthocyanins (TA) were determined by the pH differential method (AOAC Official Method 2005.02). Juices were diluted according to appropriate dilution ratios (1 part sample and 4 parts buffer) by adding both 0.025 mol/L KCl (pH 1.0) or 0.4 mol/L CH_3_COONa·3H_2_O (pH 4.5) buffer solutions (Avantor Performance Materials). Samples were mixed and left in the dark for 30 min. Absorbance was measured on a Hitachi U-1900 spectrophotometer at 520 nm and 700 nm, and the results were calculated using the following formula:

A = [(A_520_ − A_700_)_pH1.0_ − (A_520_ − A_700_)_pH4.5_] (1)

where A_520_ is the absorbance measured at 520 nm and A_700_ is the absorbance measured at 700 nm, at pH 1.0 and 4.5, respectively.

Total anthocyanins were expressed as milligrams of cyanidin-3-mono-glucoside equivalents (CGE) per liter of juice [28].

Molar extinction coefficient = 26.900 L/mol/cm and molecular weight = 449.2 g/mol. 

### 2.9. Statistical Analysis

The results were statistically analyzed by calculating the mean and standard deviation. The interpretation of the results was performed with MS Excel 2010 Analysis ToolPak software (Microsoft, Redmond, DC, USA). The conditions of normality and constant variance were satisfied, so we performed the one-way analysis of variance (ANOVA) using the Tukey’s post-hoc test: different letters in the same column indicate statistical significance (at least *p* < 0.05).

## 3. Results and Discussion

Most of the analyzed energy drinks had a typical composition, including 320 mg/Kg caffeine content. Sample C11 was an exception in that containing 480 mg/Kg of caffeine. The soluble solids content in the classic EDs was about 120–130 g/Kg, except sugar-free energy drinks (C12 and C13) (Table 1). The energy drinks with added fruit juices or natural pigments tended to have a higher content of soluble solids, by as much as 20–40 g/Kg, more than the classic EDs.

The analyzed classic EDs were characterized by low pH, ranged from 3.18 to 3.66 (Table 1). The fruit-enriched EDs had a slightly lower pH, ranged from 2.32 to 3.60. Seven of the ten fruit EDs had a pH less than 3.0. The high acidity of EDs could be caused mainly by their content of vitamin C. The classic EDs contained 75 mg/Kg to 150 mg/Kg of sample (expressed as AA). The fruit EDs had significantly (*p* ≤ 0.05) more vitamin C, from about 150 mg/Kg to over 500 mg/Kg (except samples F9 and F10; Table 2). Furthermore, the low pH of all EDs was due to the presence of acidity regulator, i.e., citric acid and/or sodium citrate. The content of these acids in EDs increased the antioxidant effect.

In our study, determination of antioxidant capacity was based on DPPH assay, which is very popular in food analyses, especially of fruit, vegetables, and juices [25,29,30]. DPPH assay is a simple and inexpensive method [23], which ensures a high linear correlation with respect to both the content of flavonoids [31] and other methods [24]. Our results showed that EDs with added fruit juices or natural pigments had the highest antioxidant capacity. The samples F2, F3, F4, and F8 were characterized by the highest antioxidant capacity ranged 240–290 mg/L (expressed as Tx) (Table 2). The remaining fruit EDs had lower values below 200 mg/L. Antioxidant capacity of classic EDs ranged typically from 60 mg/L to 90 mg/L, the highest value among them was demonstrated by sample C5 (99.0 ± 0.4 mg/L). Assay ABTS confirmed the high antioxidant capacity of fruit EDs. Obtained results for fruit EDs ranged from 120.6 mg/L to 449.0 mg/L (expressed as Tx), and for classic EDs only from 95.4 mg/L to 157.4 mg/L. We observed higher results for ABTS assay than DPPH assay. Both radicals (DPPH and ABTS) might have also various affinities to other compounds present in the samples. ABTS radical could react with flavonoids, giving slightly overstated results. The DPPH assay is more reliable and gives more accurate results [32].

Similarly to antioxidant capacity, fruit EDs had the highest total polyphenol concentration, especially F1 and F3 (703.3 ± 13.2 mg/L and 581.0 ± 5.8 mg/L, respectively; expressed as GAE) (Table 2). These ones had a large contribution of fruit juices in their composition (F3) or anthocyanins (F1), as well as guarana extract (both samples). On the other hand, classic EDs were characterized by lower polyphenols concentration, typically from 115 mg/L to about 300 mg/L. The exception in that were samples C10 and C11 (above 500 mg/L). We noted that samples C10 and C11 had guarana extract additive declared. In our studies, small quantities of total anthocyanin concentration were found in some fruit EDs (samples F1, F3, F5, F6, F8), because those beverages were added with fruit juices naturally rich in them. The highest of total anthocyanins were characterized by F1 and F3 energy drinks (35.2 ± 2.0 mg/L and 30.2 ± 2.0 mg/L, respectively; expressed as CGE). Smaller amounts of anthocyanin were found in the sample F8 (Table 2). In classic EDs, total anthocyanins were not detected.

**Figure 1:**
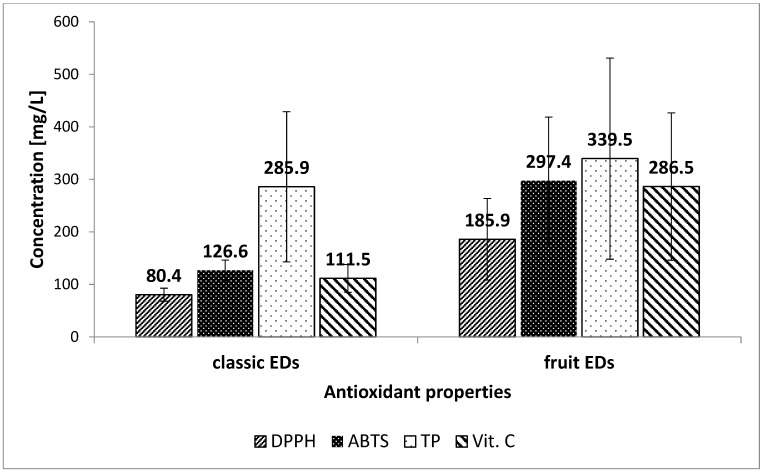
Mean values of antioxidant properties.

The mean values of antioxidant properties for fruit and classic EDs were calculated (Figure 1). Obtained results showed significantly higher values for fruit EDs. The average antioxidant capacity for fruit EDs was 185.9 mg/L, while only 80.4 mg/L (expressed as Tx) for classic EDs. Similar results were observed in the ABTS assay. The highest antioxidant capacity was characterized by fruit EDs (mean value 297.4 mg/L), while for the classic it was much lower (mean value 126.6 mg/L). Total polyphenols concentration was also higher for fruit EDs (339.5 mg/L versus 285.9 mg/L; expressed as GAE). Moreover, energy drinks with the addition of fruit juices and natural pigments had a much higher content of vitamin C (286.5 mg/Kg versus 111.5 mg/Kg; expressed as AA). Furthermore, the correlation coefficients were calculated and showed that vitamin C content had a greater impact on antioxidant capacity (*r*^2^ = 0.750) than total polyphenols concentration (*r*^2^ = 0.431).

The literature data show only few references concerning the determination of antioxidant properties of EDs. Diamantini et al. [33] reported that EDs containing extracts of guarana, ginseng, yerba mate, or caffeine presented higher total polyphenols concentration and higher antioxidant activity than EDs without additives. As reported by McLellan and Lieberman [34], guarana extract contains saponins and tannins, which exert antioxidant effects. In another study done by Witkowska et al. [35], among 11 different EDs, the highest antioxidant capacity, determined by DPPH and FRAP (ferric reducing antioxidant power), was demonstrated by samples with added fruit juices. These samples were also the richest source of polyphenolic compounds, i.e., 147 mg/L and 195 mg/L, respectively (expressed as GAE) [35]. These authors also found a lower concentration of polyphenols in the classic EDs analyzed, ranging from 60 mg/L to 90 mg/L [35]. Our research, in comparison with Witkowska et al. [35], showed a bit higher results for classic EDs, but much higher for fruit EDs (Table 2). Both studies noted no significant differences in total polyphenols between some fruit EDs and classic ones.

Compared to other food products such as plain fruit juices, the analyzed EDs were characterized by relatively poor antioxidant properties. The total polyphenols concentration and antioxidant capacity of energy drinks determined in our research were significantly lower than values which have been found in juices, nectars, and fruit drinks [25,36] or even in teas [37]. EDs should not be considered as a good source of antioxidants. Slightly better antioxidant capacity of fruit EDs may have been caused by the presence of vitamin C and polyphenols derived from the added fruit juice, or from herbal extracts (ginseng, guarana) as well as other additives, e.g., citric acid or ascorbic acid. The slightly higher antioxidant properties of fruit EDs due to the presence of antioxidant compounds could contribute to a small degree to the reduction of oxidative stress.

The results of total anthocyanins concentration showed that the highest amounts of these compounds were samples F1 and F3. The first fruit energy drink had added anthocyanins, and the second apple, black currant, grape, and strawberry juices (Table 1). Other fruit EDs contained anthocyanins because they contained apple juice (F5), apple and pomegranate juices (F6), and acai and goji juices. Classic EDs were characterized by relatively low antioxidant properties. Determined antioxidant capacity of classic EDs was a result of presence of taurine [38] and other antioxidant molecules present in classic EDs.

The analyzed energy drinks were characterized by considerable amounts of sugar in their composition. One 250 mL can of energy drink provide as much as 30 g of sugar. Thus, regular consumption of EDs poses a risk of excessive sugar ingestion, which is conducive to overweight, obesity, and tooth decay. Health risks related to large quantities of sugar in drinks have been reported by numerous researchers. Dawis et al. [39] concluded that high sugar content of EDs can increase the risk of obesity. Others have drawn attention to the dangers of developing type 2 diabetes and dental problems, including enamel erosion [40,41,42]. Clapp et al. have shown that energy drinks contain excessive amounts of free sugars, ranging from 25.5 g to 69.2 g. The authors concluded that regular consumption of energy drinks could contribute to dental erosion and the development of obesity [43]. In addition to the presence of sugar in EDs, an additional problem may be the low pH of drinks could contribute to dental erosion. In our study, the pH of EDs was from 2.32 to 3.66 and it was the lowest in fruit energy drinks. In another study, all five energy drinks investigated had pH values below the critical value (5.5) associated with dental erosion [43].

Consumption of EDs fortified with fruit juices or herbal extracts provides some antioxidants compared to classic EDs, however, it still does not solve the problems of associated health risks. EDs contain large amounts of sugar (mainly saccharose), which could contribute to overweight and obesity. Furthermore, excessive consumption of EDs may be associated with a greater risk of cardiovascular disease, due to the synergistic effect of glucose and caffeine [12,13]. Previous studies showed that consumption of energy drinks (EDs) rich in caffeine and sugar may have an adverse effect on human health, mainly by raising arterial blood pressure and blood glucose level; in the long-term, it increases the risk of cardiovascular diseases and type 2 diabetes [8,44,45,46]. The problem increases when the excessive consumption of EDs is combined with the simultaneous consumption of other sources of caffeine, such as coffee or cola-type beverages. This fact concerns especially adolescents and young adults who consume several caffeinated drinks daily. Current knowledge of health risks associated with excessive consumption of EDs is scarce. There are scientific studies that showed the beneficial effects of moderate amounts of caffeine on human health [9,10,11]. Most of these studies present data on the effects of a daily caffeine intake lower than 600 mg [9] and, moreover, concern plain coffee or extracts containing caffeine, but did not include energy drinks.

## 4. Conclusions

The research results have proven that energy drinks are not a rich source of polyphenols and do not have high antioxidant capacity. Energy drinks containing fruit juices and natural pigments (e.g., anthocyanins) show significantly higher antioxidant properties (total polyphenols concentration and antioxidant capacity) than classic EDs. They also have a higher vitamin C content. It should be noted that energy drinks, especially classic types, are a much poorer source of antioxidants than fruit juices, fruit drinks, or even teas. Moreover, the high content of caffeine and sugar means that EDs should be consumed in moderation. High sugar content and low pH, especially fruit energy drinks, could contribute to dental erosion and the development of obesity. In conclusion, fruit EDs available on the market look like an interesting alternative to classic EDs since the addition of good quality fruit juices (especially from colored berries) may increase the antioxidant capacity. Furthermore, the natural content of sugar of fruit juices could result in a reduction of saccharose or glucose-fructose syrup additives. For the reported reasons, fruit EDs may be a better choice than classic EDs.

## Figures and Tables

**Table 1:** Characteristics of the analyzed energy drinks.

Sample	Kind of ED	Standard Ingredients	Extra Ingredients	Energy Per 100 mL (Kj)	Declared Caffeine Content (mg/Kg)	Declared Sugar Content (g/Kg)	pH	Soluble Solids Content (g/Kg)
C1	classic	taurine, inositol, niacin, pantothenic acid, vitamin B_6_, B_12_, acidity regulator (sodium citrate and/or citric acid)	–	192.6	320	108	3.35 ^c^	117.5 ^b^
C2	classic		–	184.2	320	103	3.22 ^c,d^	130.0 ^b^
C3	classic		–	192.6	320	110	3.29 ^c^	125.0 ^b^
C4	classic		–	192.6	320	110	3.34 ^c^	125.0 ^b^
C5	classic		–	192.6	320	110	3.45 ^b^	125.0 ^b^
C6	classic		–	192.6	320	110	3.31 ^c^	125.0 ^b^
C7	classic		–	184.2	320	103	3.43 ^b^	120.0 ^b^
C8	classic		–	192.6	320	110	3.30 ^c^	120.0 ^b^
C9	classic		–	188.4	320	105	3.60 ^a^	117.5 ^b^
C10	classic		Guarana	192.6	320	110	3.32 ^c^	130.0 ^b^
C11	classic		guarana, glucuronolactone	188.4	480	100	3.30 ^c^	125.0 ^b^
C12	classic		–	12.6	320	<5	3.18 ^d^	20.0 ^c^
C13	classic		–	16.7	320	<1	3.48 ^b^	20.0 ^c^
C14	classic		–	188.4	320	101	3.66 ^a^	120.0 ^b^
F1	fruit	taurine, inositol, niacin, pantothenic acid, vitamin B_6_, B_12_, acidity regulator (sodium citrate and/or citric acid)	guarana, anthocyanins,	234.5	320	133	2.73 ^e^	145.0 ^a^
F2	fruit		ginseng, carotenoids, pineapple juice (3%)	192.6	320	110	2.54 ^e,f^	125.0 ^b^
F3	fruit		guarana, juices: apple (13.8%), black currant (4.3%), grape (0.9%), strawberry (0.5%), raspberry (0.5%), carrot and hibiscus concentrate	188.4	320	108	3.32 ^c^	120.0 ^b^
F4	fruit		carotenoids, ascorbic acid	192.6	320	110	3.27 ^c^	125.0 ^b^
F5	fruit		guarana, ginseng, apple, and ascorbic acids	268.0	320	150	2.84 ^e^	160.0 ^a^
F6	fruit		guarana, apple, and pomegranate juices, carrot and hibiscus concentrate	192.6	350	110	2.39 ^f^	115.0 ^b^
F7	fruit		guarana, ginseng, ascorbic acid, glucuronolactone	251.2	320	140	2.34 ^f^	150.0 ^a^
F8	fruit		guarana, ginseng, acai, and goji juices, black tea extract, glucuronolactone	41.9	320	210	3.60 ^a^	40.0 ^c^
F9	fruit		guarana, ginseng, apple, and citric acids, glucuronolactone	251.2	320	140	2.32 ^f^	145.0 ^a^
F10	fruit		carotenoids, ascorbic acid	201.0	150	130	2.80 ^e^	130.0 ^b^

Different letters in the same column indicate statistical significance (at least *p* ≤ 0.05); *n* = 3.

**Table 2:** Antioxidant properties of various energy drinks.

Sample	Vitamin C (mg/Kg)	DPPH (mg/L)	ABTS (mg/L)	TP (mg/L)	TA (mg/L)
	Classic energy drinks				
C1	107.2 ^e^	73.0 ± 0.2 ^d^	109.5 ± 1.9 ^d^	158.0 ± 2.0 ^f,g^	nd
C2	106.9 ^e^	77.0 ± 0.2 ^d^	119.3 ± 2.0 ^d^	265.0 ± 2.4 ^d,e^	nd
C3	122.4 ^d,e^	83.0 ± 0.1 ^c,d^	131.1 ± 2.2 ^c,d^	290.0 ± 4.7 ^d^	nd
C4	75.9 ^f^	89.0 ± 0.3 ^c^	142.4 ± 3.4 ^c^	252.0 ± 2.5 ^e^	nd
C5	107.6 ^e^	99.0 ± 0.4 ^c^	157.4 ± 2.5 ^c^	276.0 ± 1.8 ^d^	nd
C6	76.7 ^f^	90.0 ± 0.3 ^c^	138.6 ± 1.8 ^c^	268.0 ± 3.2 ^d,e^	nd
C7	153.5 ^d^	94.0 ± 0.1 ^c^	145.7 ± 2.1 ^c^	250.0 ± 3.9 ^e^	nd
C8	81.1 ^f^	97.0 ± 0.2 ^c^	155.2 ± 3.2 ^c^	314.0 ± 1.6 ^d^	nd
C9	141.9 ^d^	72.0 ± 0.2 ^d^	113.8 ± 1.2 ^d^	432.0 ± 1.7 ^c^	nd
C10	142.4 ^d^	83.0 ± 0.1 ^c d^	132.8 ± 1.7 ^c d^	590.0 ± 10.6 ^b^	nd
C11	143.7 ^d^	76.0 ± 0.1 ^d^	120.8 ± 1.2 ^d^	523.0 ± 4.6 ^b^	nd
C12	83.7 ^f^	60.0 ± 0.3 ^d^	95.4 ± 1.0 ^d^	115.0 ± 1.8 ^h^	nd
C13	105.8 ^e^	67.0 ± 0.2 ^d^	107.2 ± 0.8 ^d^	138.0 ± 3.4 ^g^	nd
C14	111.6 ^e^	65.0 ± 0.1 ^d^	103.3 ± 1.2 ^d^	131.0 ± 2.5 ^g^	nd
	Fruit energy drinks				
F1	–	163.0 ± 0.1 ^b^	261.0 ± 3.8 ^b^	703.3 ± 13.2 ^a^	35.2 ± 2.0 ^a^
F2	301.3 ^b^	278.0 ± 1.4 ^a^	439.2 ± 12.0 ^a^	381.0 ± 2.0 ^c^	nd
F3	–	286.0 ± 3.4 ^a^	449.0 ± 11.4 ^a^	581.0 ± 5.8 ^b^	30.2 ± 2.0 ^a^
F4	359.0 ^b^	263.0 ± 0.9 ^a^	415.5 ± 8.2 ^a^	440.0 ± 9.1 ^c^	nd
F5	230.0 ^c^	175.0 ± 1.1 ^b^	278.2 ± 4.0 ^b^	202.0 ± 1.3 ^f^	9.6 ± 1.0 ^c^
F6	–	94.0 ± 0.4 ^c^	152.2 ± 2.0 ^c^	139.0 ± 2.6 ^g^	6.8 ± 1.0 ^c^
F7	520.2 ^a^	168.0 ± 0.5 ^b^	277.2 ± 4.2 ^b^	239.0 ± 4.4 ^e^	nd
F8	–	242.0 ± 1.0 ^a^	392.0 ± 7.2 ^a^	316.0 ± 3.5 ^d^	16.4 ± 1.0 ^b^
F9	155.1 ^d^	74.0 ± 0.3 ^d^	120.6 ± 1.9 ^d^	105.0 ± 5.4 ^h^	nd
F10	153.1 ^d^	116.0 ± 0.1 ^c^	189.1 ± 3.1 ^c^	289.0 ± 11.2 ^d^	nd

Data are mean ± standard deviation (*n* = 5). Different letters in the same row indicate statistical significance (at least *p* ≤ 0.05). Vitamin C content is expressed as milligrams of ascorbic acid; DPPH—antioxidant activity measured by DPPH method (expressed as milligrams of Trolox equivalents); ABTS—antioxidant activity measured by ABTS method (expressed as milligrams of Trolox equivalents); TP—total polyphenols measured by Folin–Ciocalteu assay (expressed as milligrams of gallic acid equivalents); TA—total anthocyanins measured by the pH differential method (expressed as milligrams of cyanidin-3-glucoside equivalents); nd—non detected.
